# Dural ectasia: a manifestation of type 1 neurofibromatosis

**DOI:** 10.11604/pamj.2018.31.226.9797

**Published:** 2018-12-07

**Authors:** Ilyas Derdabi, Hajar El Jouadi, Meryem Edderai

**Affiliations:** 1 Department of Radiology, Hospital Mohammed V, Rabat, Morocco

**Keywords:** Dural ectasia, neurofibromatosis type 1, anterior meningocele

## Abstract

Dural ectasia denotes circumferential expansion or dilatation of the dural sac, and has been frequently reported in association with type 1 neurofibromatosis (NF1). The pathogenesis has not been defined, but its correlation with NF1 infers a congenital malformative hypothesis. The neural elements in the dilated sleeve typically are not enlarged or abnormal, nevertheless the enlarged area contain an increased amount of cerebrospinal fluid. The dura in the area of ectasia is extremely thin and fragile, and erodes the surrounding bony structures destabilising the spine and permitting spectacular spinal deformities. We present a case: a 40-year-old woman suffering from neurofibromatosis type 1 who developed a thoracic dural ectasia and anterior meningocele.

## Introduction

Dural ectasia, which is often idiopathic, is seen both in patients with neurofibromatosis and Marfan’s syndrome. In neurofibromatosis, the ectasia is most often seen in the thoracic region as posterior mediastinal masses. We present a case of a 40-year-old woman suffering from neurofibromatosis type 1 who developed dural ectasia and anterior meningocele.

## Patient and observation

**Figure 1:**
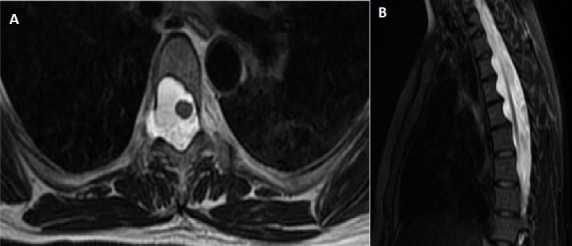
sagittal (A) and axial (B) T2-weighted spin-echo magnetic resonance image of the thoracic spine showing posterior vertebral scalloping by dural ectasia

**Figure 2:**
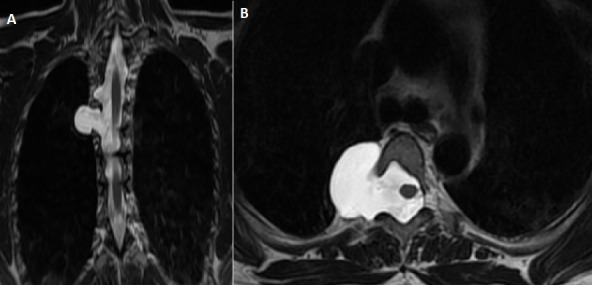
coronal (A) and axial (B) T2-weighted spin-echo magnetic resonance image of the dorsal spine showing anterior-meningocele D3-D4

A 40 year-old woman was admitted to the hospital with low back pain. She was known of having type 1 neurofibromatosis. There was no history of trauma. The neurological examination was normal. MRI of the cervico-thoraco-lumbar spine showed an enlargement of dural sac and root sheaths, extended about 16 centimeter from D2 to D7, thus responsible of foramens enlargement and posterior vertebral scalloping (Figure 1). There was an extra-foraminal extension from D3 to D4 providing a dorsal anterior-meningocele (Figure 2). There was no abnormal medullary signal.

## Discussion

Dural ectasia, widening of the dural sac, is associated with the dystrophic changes found in type 1 neurofibromatosis and appears as dural outpouchings (meningoceles) through enlarged neuroforamina, leading to erosion of surrounding bone and displacement of adjacent organs. Up to 70% – 80% of dural ectasia is found in patients with type 1 neurofibromatosis [1], although it is also associated with Marfan syndrome (in up to 92%) [2], Ehlers-Danlos syndrome, ankylosing spondylitis and achondroplasia, and can occur idiopathically [3]. Meningioma and spinal nerve fibroma are associated with type 1 neurofibromatosis, and are also capable of protruding laterally through and eroding the neuroforamina.

Classic radiologic findings include erosion of the central posterior vertebral body, wedging and posterior scalloping of the vertebral body, pedicle erosion, foraminal enlargement and kyphosis. The complications of progressive vertebral body erosion include angular deformities (usually fewer than six vertebral levels) and vertebral fractures and dislocations. Neurologic deficit rarely occurs because the spinal canal is widened [4]. These deformities have been known to progress and evolve during the course of disease, a phenomenon known as “modulation [5]. Early surgical stabilization may be necessary to prevent severe deformity and late complications.

## Conclusion

Within the CNS, NF-1 manifests as a weakness of the dura, which is the tough covering of the brain and spine. Weakness of the dura leads to focal enlargement terms dural ectasia due to chronic exposure to the pressures of CSF pulsation.

## Competing interests

The authors declare no conflict of interest.

## References

[1] Gajeski BL, Kettner NW, Awwad EE (2003). Neurofibromatosis type I: clinical and imaging features of von Recklinghausen’s disease. J Manipulative Physiol Ther.

[2] Fattori R, Nienaber CA, Descovich B (1999). Importance of dural ectasia in phenotypic assessment of Marfan’s syndrome. Lancet.

[3] Toyoda K, Taguchi T, Kaneko K (2005). High-grade L5 spondylolisthesis associated with dural ectasia in neurofibromatosis. J Orthop Sci.

[4] De Kleuver M, van Jonbergen JPW, Langeloo DD (2004). Asymptomatic massive dural ectasia associated with neurofibromatosis type 1 threatening spinal column support: treatment by anterior vascularized fibular graft. J Spinal Disord Tech.

[5] Durrani AA, Crawford AH, Chouhdry SN, Saifuddin A, Morley TR (2000). Modulation of spinal deformities in patients with neurofibromatosis type 1. Spine (Phila Pa 1976).

